# Modulation of Cortical Oscillations by Low-Frequency Direct Cortical Stimulation Is State-Dependent

**DOI:** 10.1371/journal.pbio.1002424

**Published:** 2016-03-29

**Authors:** Sankaraleengam Alagapan, Stephen L. Schmidt, Jérémie Lefebvre, Eldad Hadar, Hae Won Shin, Flavio Frӧhlich

**Affiliations:** 1 Department of Psychiatry, University of North Carolina at Chapel Hill, Chapel Hill, North Carolina, United States of America; 2 UNC/NCSU Joint Department of Biomedical Engineering, University of North Carolina at Chapel Hill, Chapel Hill, North Carolina, United States of America; 3 Krembil Research Institute, University Health Network, Toronto, Ontario, Canada; 4 Department of Mathematics, University of Toronto, Toronto, Ontario, Canada; 5 Department of Neurosurgery, University of North Carolina at Chapel Hill, Chapel Hill, North Carolina, United States of America; 6 Department of Neurology, University of North Carolina at Chapel Hill, Chapel Hill, North Carolina, United States of America; 7 Neurobiology Curriculum, University of North Carolina at Chapel Hill, Chapel Hill, North Carolina, United States of America; 8 Department of Cell Biology and Physiology, University of North Carolina at Chapel Hill, Chapel Hill, North Carolina, United States of America; 9 Neuroscience Center, University of North Carolina at Chapel Hill, Chapel Hill, North Carolina, United States of America; Radboud University Nijmegen, NETHERLANDS

## Abstract

Cortical oscillations play a fundamental role in organizing large-scale functional brain networks. Noninvasive brain stimulation with temporally patterned waveforms such as repetitive transcranial magnetic stimulation (rTMS) and transcranial alternating current stimulation (tACS) have been proposed to modulate these oscillations. Thus, these stimulation modalities represent promising new approaches for the treatment of psychiatric illnesses in which these oscillations are impaired. However, the mechanism by which periodic brain stimulation alters endogenous oscillation dynamics is debated and appears to depend on brain state. Here, we demonstrate with a static model and a neural oscillator model that recurrent excitation in the thalamo-cortical circuit, together with recruitment of cortico-cortical connections, can explain the enhancement of oscillations by brain stimulation as a function of brain state. We then performed concurrent invasive recording and stimulation of the human cortical surface to elucidate the response of cortical oscillations to periodic stimulation and support the findings from the computational models. We found that (1) stimulation enhanced the targeted oscillation power, (2) this enhancement outlasted stimulation, and (3) the effect of stimulation depended on behavioral state. Together, our results show successful target engagement of oscillations by periodic brain stimulation and highlight the role of nonlinear interaction between endogenous network oscillations and stimulation. These mechanistic insights will contribute to the design of adaptive, more targeted stimulation paradigms.

## Abstract 2

This study presents mathematical models that explain the effect of temporally patterned electrical stimulation on cortical oscillations and provides supporting evidence using data recorded directly from human cortex during transcranial electrical stimulation.

## Author Summary

Rhythms in the brain are believed to play an important role in cognition. Disruptions in these oscillations are associated with a number of neurological and psychiatric disorders. Therefore, noninvasive brain stimulation techniques that target these oscillations offer promise as therapeutic tools. In particular, transcranial alternating current stimulation (tACS) applies a periodic stimulation waveform to engage specific oscillations in the cortex. Although recent studies provide evidence for the modulation of cortical oscillations by tACS, the exact mechanism by which the effects are produced is poorly understood. We propose two mathematical models of interaction between periodic electrical stimulation and ongoing brain activity that may explain the effects of tACS. In addition, we present a unique dataset in which we stimulated the patients’ cortical surface with subdural electrodes and observed the responses to stimulation in neighboring electrodes. We found that stimulation enhanced ongoing oscillations both during and immediately after stimulation. This enhancement depended on the brain state, thereby supporting our proposed models. Our results demonstrate the effect of electrical stimulation on cortical oscillations and highlight the importance of considering the state of the brain when designing electrical stimulation therapies for disorders of the central nervous system.

## Introduction

Oscillations in a wide range of frequencies represent a ubiquitous organizational pattern of cortical network dynamics [1]. In particular, oscillations in the alpha frequency band are pronounced activity patterns routinely observed in posterior leads of electroencephalograms (EEGs) of healthy human participants with closed eyes [2]. This alpha rhythm was originally considered to reflect an “idling” state of cortex in the absence of sensory input [3]. However, it has become clear that alpha oscillations dynamically regulate processing of sensory input and mediate long-range cortical interaction dynamics [4,5]. These roles of alpha oscillations in cognition and behavior have been demonstrated by the use of noninvasive brain stimulation to modulate alpha oscillations. Repetitive transcranial magnetic stimulation (rTMS) in the alpha frequency range modulates sensory detection [6,7], likely by entraining alpha oscillations. Similarly, transcranial alternating current stimulation (tACS), which applies a weak, sine-wave electric current to the scalp, alters cognitive processes by targeting alpha oscillations [8–10]. In addition to the causal role of alpha oscillations in mediating behavior, oscillations in this frequency band are selectively impaired in psychiatric illnesses such as major depressive disorder, autism, and schizophrenia [11–13]. As a result, alpha oscillations have recently emerged as a potential target for therapeutic intervention with noninvasive brain stimulation. The Food and Drug Administration (FDA) has approved rTMS with 10 Hz stimulation frequency as a treatment for major depressive disorder [14]. Of note, the 10 Hz patterning was the result of an attempt to develop rTMS paradigms with outlasting effects, and considerations of the possible effect on alpha oscillations emerged later [15]. Furthermore, other noninvasive stimulation modalities that target alpha oscillations for the treatment of major depressive disorder are under active investigation [16–18].

Despite the confluence of evidence for modulation of alpha oscillations and promising clinical applications, the network mechanisms by which brain stimulation modulates alpha oscillations have remained unclear. Therefore, computational modeling efforts have remained limited. In contrast, both low frequency (<4 Hz) and fast cortical oscillations have been investigated as a target of weak periodic perturbations. Both computer simulations [19–21] and slice experiments [22,23] have provided mechanistic hypotheses on the interaction dynamics of endogenous activity and stimulation. First, periodic stimulation matched to the endogenous frequency allows weak stimulation amplitudes to entrain endogenous oscillation [19]. Second, stimulation frequencies that differ from the endogenous frequency cause enhancement of oscillation power at the endogenous frequency in case of high-amplitude endogenous oscillations [23]. Yet, it remains unclear if and how these principles apply to stimulation that targets alpha oscillations, which are of thalamo-cortical origin [24–26]. In humans, concurrent tACS and EEG showed enhanced activity at the stimulation frequency (10 Hz) possibly caused by entrainment of endogenous alpha oscillators [9]. Also, stimulation at individual alpha frequencies produced enhancement when the participants had their eyes open, but not when the participants had their eyes closed [27]. In contrast, brief epochs of tACS failed to cause entrainment determined by a lack of phase synchronization [28,29], suggesting plasticity as an alternative mechanism for the observed increase in oscillation power. Thus, there is no clear consensus on the mechanism underlying the effects of periodic brain stimulation that targets alpha oscillations [15].

To fill this gap, we aimed to develop models that would help elucidate the effect of periodic stimulation on alpha oscillations and demonstrate the experimental validity of the models using neurophysiological effect of stimulation in the alpha frequency in human cortex. The combination of direct cortical stimulation with electrocorticography (ECoG) serves as an ideal approach, since it allows for the simultaneous manipulation and recording of oscillations at a finer spatial scale. We derived our approach from the direct cortical stimulation with high stimulation amplitude and frequency, which is clinically used during invasive monitoring to determine “eloquent cortex” for surgical planning in patients with pharmacoresistant epilepsy [30,31].

Here, we developed a simple summation model to explain the state-dependent effects of stimulation and a network model to explain the outlasting effects of stimulation. The nature of periodic perturbation was chosen to match the stimulation used in the ECoG experiment. We applied 10 Hz periodic pulse stimulation and recorded directly from the parietal region of the brain in three patients. Grid electrodes for electrocorticography had been implanted in these patients before resective epilepsy surgery.

**Fig 1:**
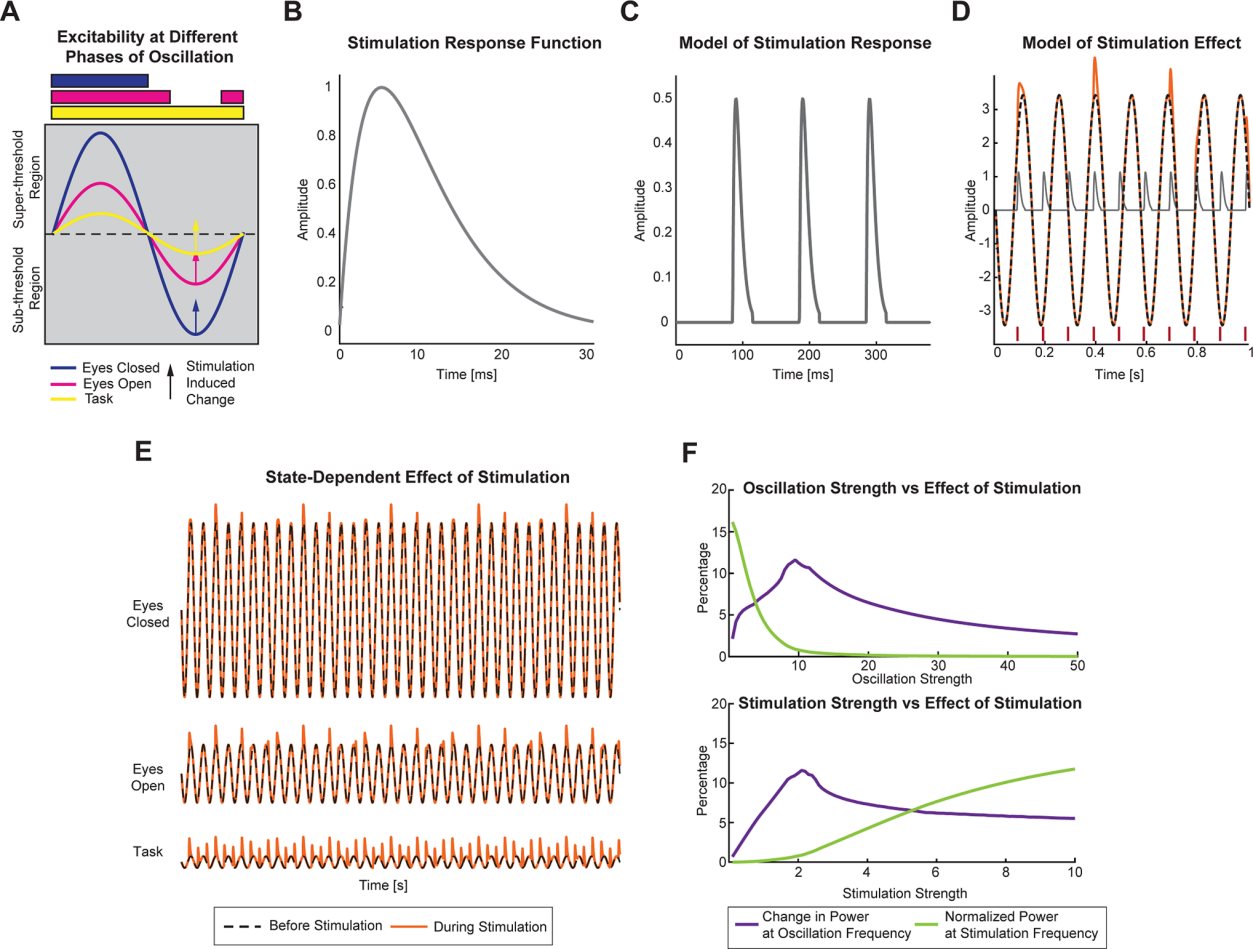
Simple Static Model Explains State-Dependence. (A) The endogenous oscillation is denoted as a pure sine wave, and the amplitude of the sine wave represents the strength of oscillation in the three states studied here. Dotted line denotes the threshold above which stimulation produces a change (denoted by arrow). When the change produced by stimulation is below the threshold, no change in the oscillation is observed. However, when the threshold is crossed, stimulation produces a response that decays with time. The bands denote the phases of the oscillation at which stimulation produces a change in the oscillation. For stronger oscillations (eyes-closed), the phases at which stimulation produces change are minimal. For very weak oscillations (task-engaged), stimulation produces change at all phases. (B) The function used to model the stimulation response. The stimulation response is modeled as the linear convolution between the stimulation pulse and this response function. (C) Convolution between stimulation pulses and stimulation response function. (D) Example traces illustrating the model behavior. The red lines at the bottom denote the timing of the stimulation pulses. The gray line denotes the stimulation response that is added to the oscillation waveform (black dashed line) to produce the stimulation effect (orange solid line). (E) Example traces produced using the model described in (A). In the eyes-closed state, stimulation-induced changes are minimal. In the eyes-open state, the stimulation-induced change is still constrained by the endogenous frequency. In the task-engaged state, stimulation produces change at all phases resulting in entrainment. Black dashed line represents the endogenous oscillation. Red solid line represents the waveform resulting from the addition of the stimulation waveform to the endogenous oscillation. (F) Top: Effect of varying endogenous oscillation strength. The change in power at the endogenous frequency increases until a certain limit and then decreases when the strength of oscillation relative to stimulation strength is high (violet line). The power at stimulation frequency (green line) decreases with increasing oscillation strength as stimulation effect is observed in a restricted range of phases. Bottom: Effect of varying stimulation strength for a given oscillation strength. As stimulation strength increases, the power at oscillation frequency increases until the strength relative to oscillation strength is high enough to cause increase in power at stimulation frequency. Beyond this, the increase in power at oscillation frequency is minimal, and power at stimulation frequency increases monotonically. The data shown in this figure are available online: http://dx.doi.org/10.5281/zenodo.45811

## Results

The first model we developed was inspired by the finding that alpha oscillations reflect periodic modulation of cortical excitability [32–34]. We hypothesized that the strength of the oscillation (quantified by the amplitude) determines the excitability, and that only stimulation pulses that coincide with the excitable intervals (as determined by alpha oscillation phase) would produce a change in the ongoing oscillation. We modeled ongoing oscillation as a sine wave (Fig 1A) and the stimulation response as a linear summation of this sine wave and the cortical response to pulse stimulation. The cortical response was modeled to be the convolution between the pulse train and a response function *f*(*t*) = *t*.*e* ^−0.01*t*^ (t denotes time in milliseconds; Fig 1B and 1C). The response function was motivated by the time course of response (direct and indirect) of neurons to stimulation. To account for our hypothesis, we required the response to cross a threshold (denoted by dotted horizontal line in Fig 1A) before including it in the stimulus response. As a result, the phases of the sine wave at which stimulation induced a response were restricted by the amplitude of the sine wave, as denoted in Fig 1D. The black dotted line denotes the ongoing oscillation, while the orange solid line denotes the effect of stimulation on the oscillation. The red lines at the bottom denote the times at which pulses were delivered, and the gray line denotes the neural response to the pulse train. The frequency of the oscillation was set at 7 Hz, and the stimulation frequency was set at 10 Hz. The orange line is a linear summation of the black and gray lines, subjected to the condition that only summation values greater than 0 are incorporated in the orange line. In situations in which the summation failed to reach 0, the values of the black line were used. With this condition, it can be seen in Fig 1D that only pulses that coincided with phases in which the amplitude of the oscillation is greater than 0 produced a change in the oscillation (e.g., pulses close to 0.1 s, 0.4 s, and 0.7 s).

Eyes-closed state was modeled as a state with oscillations of large amplitude, as alpha oscillations are known to be more pronounced in this state. In this case, the phases at which stimulation produced a response were most heavily constrained due to the threshold condition (denoted by a blue band in Fig 1A). Also, the relative change in amplitude as a result of the response to stimulation was minimal, given the large amplitude of the oscillation (Fig 1E). The eyes-open state was modeled as a state with oscillations of intermediate alpha oscillations. Although the strength of the oscillation was weakened in comparison to the eyes-closed state, the phases at which stimulation can induce a response were still somewhat restricted (magenta band in Fig 1A), such that the effect of stimulation was an enhancement of the endogenous oscillation (Fig 1E). The task-engaged state was modeled as the state with lowest oscillation amplitude, as task-engagement is often associated with a marked suppression of the power of alpha oscillations. In this last case, stimulation was able to induce a response at all phases (yellow band in Fig 1A), hence increasing the power at stimulation frequency (Fig 1E) and causing entrainment [35]. Therefore, this model can explain the enhancement at the endogenous frequency instead of the stimulation frequency in the presence of a sufficiently strong endogenous oscillation. We parameterized this simple static model by oscillation strength (relative amplitude of the sine-wave) and stimulation strength (relative amplitude of the response function). We found that the normalized power at the stimulation frequency strongly and monotonically depended on these two parameters (Fig 1F bottom). Enhancement at the stimulation frequency requires low oscillation strength and high stimulation strength. In contrast, power at the endogenous oscillation frequency depended less on these parameters as long as the oscillation and stimulation strength exhibited some minimal value. Of particular note, the enhancement of power at the oscillation frequency as a function of the two parameters was nonmonotonic. For example, the strongest enhancement occurred for an intermediate level of oscillation strength, since, for high levels of oscillation strength, the relative contribution of the stimulation was limited to a small range of oscillation phases in which the response of the perturbation reaches threshold.

**Fig 2:**
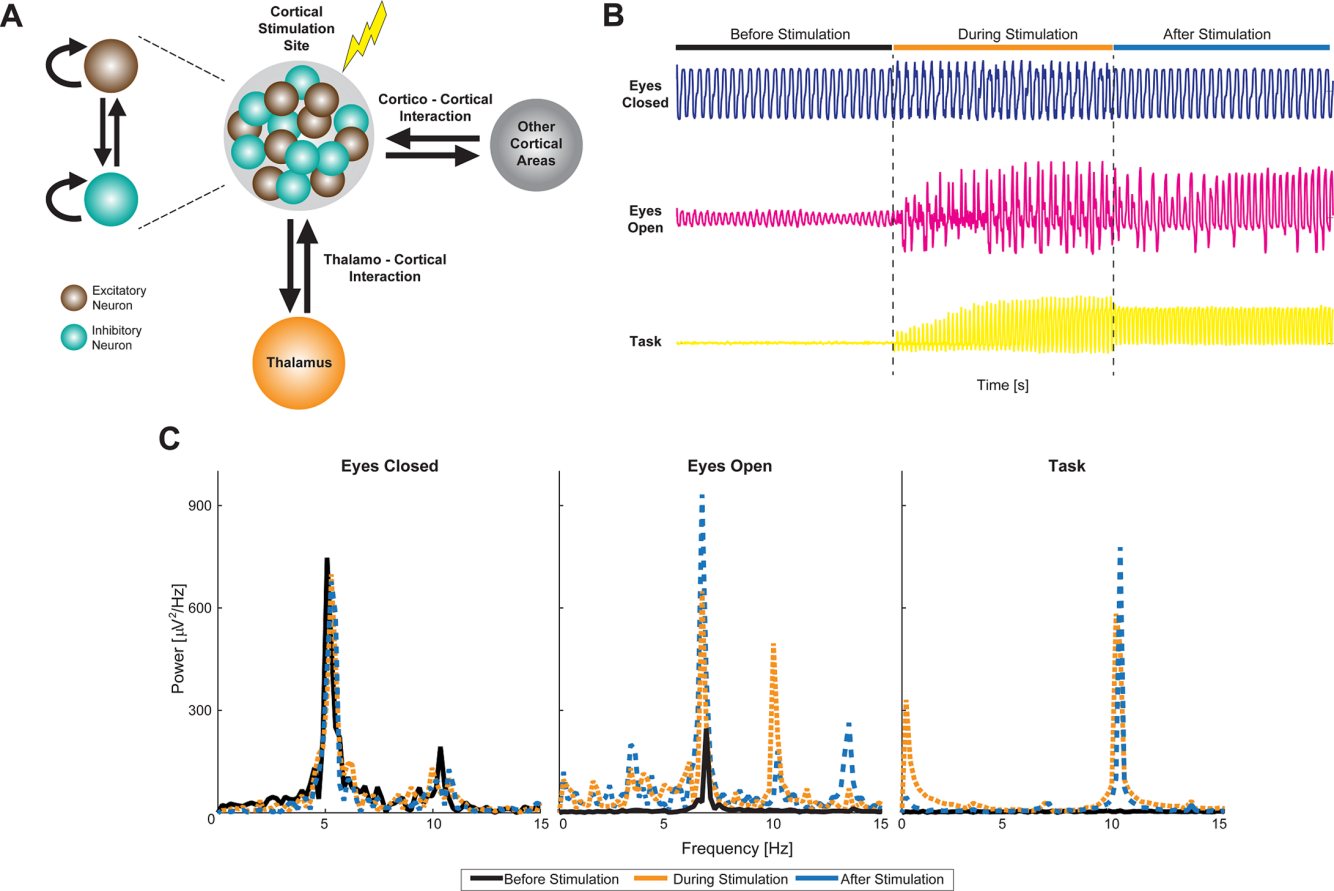
Computational Model Explains Outlasting Effects of Periodic Stimulation. (A) Schematic of computational model that includes the different components and interactions among them. The region of the cortex being stimulated is denoted by a gray circle encompassing excitatory and inhibitory neurons. The model neurons exhibit reciprocal as well as recurrent connections. The cortico-thalamic and cortico-cortical interactions are also modeled to be reciprocal. (B) Membrane potential observed from excitatory neurons in the model shows task-dependent differences in stimulation effect. During the eyes-closed state (blue trace), a strong oscillation is observed in the alpha frequency range. Stimulation onset does not alter the dynamics significantly. In the eyes-open state (magenta trace), an oscillation is still observed in the alpha frequency range. However, the strength is decreased compared to the eyes-closed state. Stimulation onset causes the amplification of this oscillation, which persists after stimulation offset and then slowly decays. In the task-engaged state (yellow trace), no strong oscillation is observed due to the external inputs that model task-related input. In this state, stimulation causes the network to oscillate at the stimulation frequency, which persists and decays in the epoch after stimulation. (C) Spectral analysis of membrane potential reveals state-dependent effect of stimulation. Refer to S1 Fig for dynamics in the absence of cortico-thalamic and cortico-cortical interactions. The data shown in this figure are available online: http://dx.doi.org/10.5281/zenodo.45811

Establishing outlasting effects of stimulation is crucial for noninvasive brain stimulation approaches to be translated from basic science research to clinical applications. The minimalistic mathematical model (Fig 1) explained the instantaneous effects of stimulation but did not provide insight into what may cause experimentally observed outlasting effects. We hypothesized that the outlasting effects are the result of multistable dynamics caused by recurrent neuronal loops. To test this hypothesis, we built a reduced network-level computational model to examine if we could reproduce the main experimental effects without including any plasticity mechanism. In particular, we aimed to reproduce (1) outlasting enhancement of alpha oscillations and (2) state-dependent stimulation effects. To this end, we performed numerical simulations of a cortical oscillator network model subjected to external stimulation and investigated its responses during three different simulated states: eyes-open, eyes-closed, and task-engaged. The model included (1) a thalamo-cortical loop as the main circuit involved in the generation and maintenance of alpha oscillations and (2) a long-range cortico-cortical loop to replicate the distributed topography of ECoG with grid electrodes (Fig 2A). The network model included the essential features of thalamo-cortical networks and yet was simplistic enough to provide insights about what mechanism could underlie the state-dependent and outlasting effects observed in the data. The eyes-closed, eyes-open, and task-engaged states were defined as states of increasing afferent inputs. The input to the excitatory neurons in the eyes-closed and eyes-open condition was assumed to be minimal; as a result, neural oscillatory dynamics were dominated by local recurrent interactions in cortex and feedback interactions in the thalamo-cortical loop. In the task-engaged condition, however, the input to the excitatory neurons was increased to represent their recruitment and engagement in the task-related cognitive processes.

Using this modeling strategy, we found that task-dependent input to the neurons acted as a gain control mechanism. Specifically, during the eyes-closed condition, the endogenous oscillations were too robust for modulation of the spectral peak frequency (Fig 2B, top trace): self-sustained, high-amplitude, alpha-like oscillations governed the network dynamics. Tightly stabilized in an oscillatory attractor, the neurons were fully driven by the feedback inputs conveyed by the thalamo-cortical loop (no alpha oscillations were observed in absence of a thalamic model component, S1A Fig), which effectively masked the drive provided by the stimulation at 10 Hz. On the other hand, in the eyes-open condition, amplification of endogenous oscillations occurred due to stimulation-induced recruitment of cortico-cortical feedback, which, in turn, also mediated the sustained effect after termination of stimulation. Indeed, our model predicts that damped oscillations at the endogenous frequency were amplified through cortical feedback loops and gave rise to the post-stimulation dynamics observed in the eyes-open condition. We found enhancement both at the endogenous frequency and at the stimulation frequency. Lastly, during the task-engaged state, increased input to the excitatory neuronal population destabilized the local oscillatory attractor, suppressing endogenous alpha rhythmic activity and making the neurons more susceptible to the stimulation. As a result, the stimulation was more effective, and a clear spectral signature of the stimulation became apparent (Fig 2B, bottom trace). Cortico-cortical feedback, recruited by the stimulation, provided the basis for the outlasting effect of stimulation (spectra in Fig 2C). Our model predicts that the maintenance of stimulation-induced dynamics after stimulation termination was due to reverberation of input-driven responses through cortical recurrence and not to resonance. Our computational model suggests that the effect of exogenous stimulation depends on the robustness of the ongoing oscillatory cortical dynamics, whereas task-related inputs tune the network to enhance susceptibility to perturbations and, therefore, enable frequency-matched responses to brain stimulation. Therefore, no synaptic plasticity was required to qualitatively reproduce state-dependent enhancement of oscillations that outlasted the stimulation.

**Fig 3:**
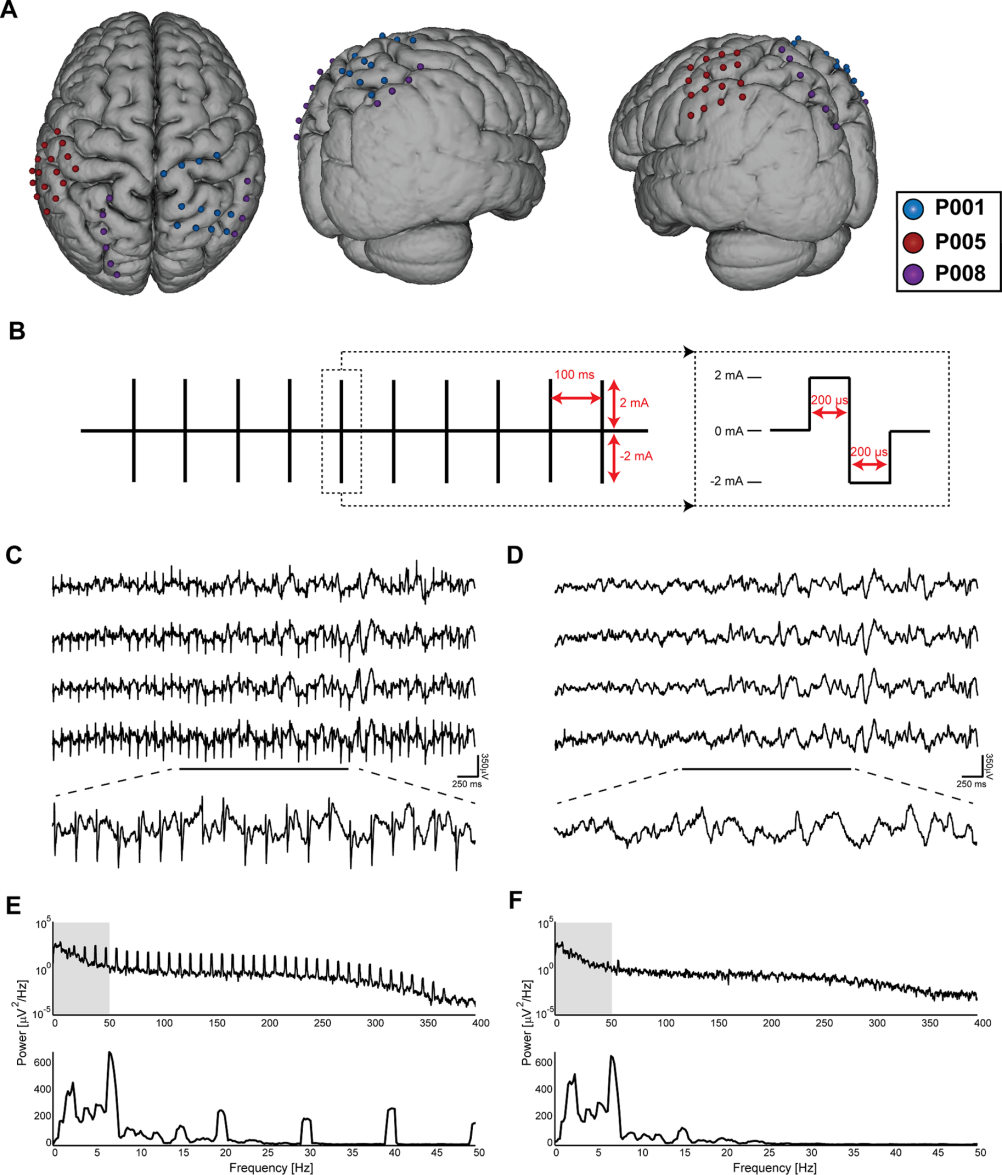
Electrode Locations and Artifact Suppression. (A) Surface model of an atlas brain showing locations of electrodes over the parietal regions in each of the three patients. Signals measured from only these electrodes were used in the analysis. Refer to S2 Fig for the stimulation electrodes and electrodes over other regions. (B) Schematic of stimulation waveform used. Stimulation consisted of one biphasic pulse 400 μs in duration every 100 ms for 5 s. (C) Stimulation artifacts in representative sample traces from four electrodes (top). Enlarged portion denoted by the black line (bottom). Artifacts appear as periodic sharp deflections with stereotyped waveforms. (D) Traces from the same four electrodes as in (B) after the artifact suppression procedure. The artifacts are suppressed compared to the signal amplitude. (E) Spectrum of the fourth waveform in (B) showing peaks at 10 Hz and harmonics of 10 Hz corresponding to artifact waveform. Top: Full frequency range. Bottom: Zoom-in on low frequencies. (F) Spectra of the same waveform after artifact suppression confirming the effectiveness of the algorithm. The only remaining exogenous peak is at 60 Hz, caused by electric line noise.

To validate the observations from the models described above, we performed intracranial stimulation experiments in epilepsy participants with subdural electrodes implanted for surgical planning. We applied 2 mA biphasic current pulse (200 μs per phase) trains of 10 Hz frequency for 5 s to the cortical surface through pairs of neighboring ECoG electrodes (Fig 3A and 3B). We simultaneously recorded ECoG signals on all other electrodes during three different conditions (eyes closed, eyes open, and task-engaged; see Materials and Methods for details) that altered cortical dynamics determined by the strength of alpha-band activity. We divided stimulation trials into 15 s segments comprised of a 5 s epoch before stimulation onset, a 5 s epoch during stimulation, and a 5 s epoch after stimulation offset. Stimulation artifacts were removed using a template matching algorithm (Fig 3C–3F; see Materials and Methods).

**Fig 4:**
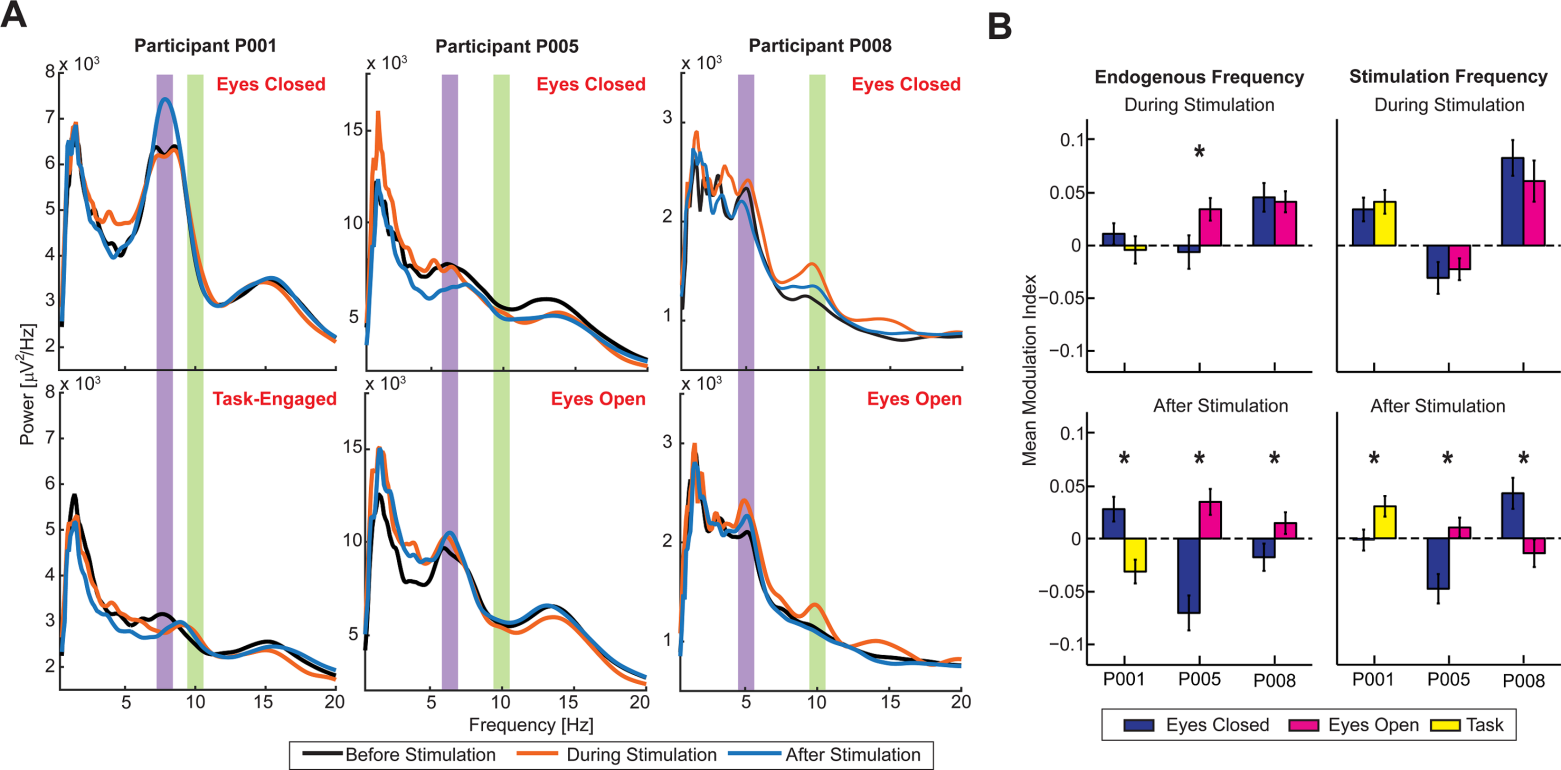
State-Dependent Modulation by Periodic Stimulation. (A) Power spectra in the epochs before stimulation (black trace), during stimulation (orange trace), and after stimulation (blue trace) for the three participants during the different states. Participant P001’s spectra showed no appreciable change in the eyes-closed state in the endogenous frequency band (violet shaded region) and minimal change in the stimulation frequency band (green shaded region) in eyes-closed state during stimulation. However, there was a change in dominant oscillation frequency from endogenous frequency to stimulation frequency in the task-engaged state. P005 showed no change in power at endogenous frequency in the eyes-closed state, while there was an increase in the eyes-open state. Power at stimulation frequency was decreased in both states. P008 showed an increase in both states in the endogenous and stimulation frequencies. (B) Mean modulation indices at the endogenous frequency (left) and the stimulation frequency (right) in the epochs during stimulation (top) and after stimulation (bottom) for each of the three participants. Bars denote standard error of mean. * denotes statistical significance (*p* < 0.05) from a paired *t* test. The data shown in this figure are available online: http://dx.doi.org/10.5281/zenodo.45811

Power spectral densities were computed in the three epochs, and modulation of power at the endogenous frequency and the stimulation frequency were quantified. A modulation index (MI) was defined as the difference in power at specific frequency bands between two epochs normalized by the sum of the power at the frequency bands in the two epochs. Fig 4A shows the average power spectra in the three epochs for each of the three participants under two different conditions, while Fig 4B shows a summary of the modulation indices at the endogenous and stimulation frequency bands for the three participants.

In three participants, we found spectral peaks that we interpreted as a sign of the dominant endogenous oscillation. In participants P001 and P005, the stimulation frequency (10 Hz) did not match the endogenous frequency. For patient P008, the peak we identified was below the classical alpha frequency, but there was an associated peak at the first harmonic frequency that approximately matched the stimulation frequency. Given this mismatch in frequencies, we considered the power at the endogenous frequency and the power at the stimulation frequency separately in our analysis. The results from participants P001 and P005 allowed verifying both the static and network models directly, as the stimulation frequency was different from the endogenous frequency or harmonics of the endogenous frequency. We found that changes in power at stimulation frequency as well as at endogenous frequency were state-dependent. Oscillation power at endogenous frequency increased in the eyes-open and eyes-closed states compared to the task-engaged state, while oscillation power at stimulation frequency increased in the task-engaged state compared to the eyes-open and eyes-closed states. In the case of participant P008, in contrast, the stimulation frequency matched the endogenous frequency at its first subharmonic. This prevented us from testing the model, as strict separation of stimulation and endogenous frequency was not possible. In this case, we found similar enhancement of power at both the endogenous and the stimulation frequencies during stimulation.

Participant P001 completed the eyes-closed and working-memory tasks. Alpha oscillations were successfully enhanced both during and after stimulation. During stimulation, activity was enhanced at the stimulation frequency of 10 Hz (eyes closed: MI = 0.034 ± 0.011; *p* = 0.002; task: MI = 0.0414 ± 0.011; *p* <0.001; one sample *t* tests) but not at the endogenous frequency (eyes closed: MI = 0.011 ± 0.01; *p* = 0.275; task: MI = -0.0042 ± 0.013; *p* = 0.747). We found no difference in power modulation as a function of state (endogenous frequency: *p* = 0.354, stimulation frequency: *p* = 0.650). However, in only the task-engaged state, there was an acceleration of oscillation frequency from the endogenous peak (7.1 Hz) toward 10 Hz with a median peak frequency at 9 Hz (*p* = 0.010; Wilcoxon rank-sum test). This change in oscillation frequency was evident in the time-domain signal and was confirmed not to be the result of stimulation artifacts (S3 Fig). Interestingly, in the epoch after stimulation, we found state-dependent modulation of oscillation power (endogenous frequency: *p* < 0.001; stimulation frequency: *p* = 0.022). After stimulation, the peak at the endogenous frequency was enhanced in the eyes-closed state (MI = 0.0279 ± 0.0117; *p* = 0.018) but decreased in the task-engaged state (MI = -0.031 ± 0.011; *p* = 0.006). In conjunction with the shift of the alpha frequency toward the stimulation frequency during the task-engaged state, the peak at the stimulation frequency was increased in the task-engaged state (MI = 0.030 ± 0.010; *p* = 0.002) but not in the eyes-closed state (MI = -0.0017 ± 0.010; *p* = 0.867). The shift in peak frequency persisted at a median of 8.95 Hz (*p* = 0.014) in the task-engaged state. In summary, the results from participant P001 confirmed the modulation of alpha oscillations by stimulation at 10 Hz both during and after stimulation. A pronounced state-dependent effect was observed after stimulation; the stimulation boosted power at the endogenous frequency or at the stimulation frequency as a function of state.

Participant P005 completed the eyes-open and eyes-closed tasks. Endogenous oscillation power was successfully enhanced only in the eyes-open state both during and after stimulation (Refer to S4 Fig for an example electrode). This enhancement was limited to the power at the endogenous frequency both during stimulation (eyes-open: MI = 0.034 ± 0.011; *p* = 0.002; eyes-closed: MI = -0.006 ± 0.016; *p* = 0.693; one sample *t* tests; difference: *p* = 0.04; two sample *t* test) and after stimulation (eyes-open: MI = 0.035 ± 0.012; *p* = 0.006; eyes-closed: MI = -0.071 ± 0.016; *p* < 0.001; difference: *p* < 0.001). The power at the stimulation frequency either decreased or did not change both during stimulation (eyes-open: MI = -0.023 ± 0.010; *p* = 0.031; eyes-closed: MI = -0.031 ± 0.015; *p* = 0.043) and after stimulation (eyes-open: MI = 0.010 ± 0.001; *p* = 0.280; eyes-closed: MI = -0.048 ± 0.014; *p* < 0.001).

Participant P008 completed the eyes-open and eyes-closed tasks. Endogenous oscillation power was also successfully enhanced in this patient. During stimulation, the power increased at the endogenous frequency (eyes-closed state: MI = 0.046 ± 0.014; *p* = 0.001; eyes-open state: MI = 0.041 ± 0.010; *p* < 0.001, one sample *t* tests; difference: *p* = 0.802; two sample *t* test) and increased again at the stimulation frequency (eyes-closed state: MI = 0.083 ± 0.017; *p* < 0.001; eyes-open state: MI = 0.061 ± 0.020; *p* = 0.002; difference: *p* = 0.397). After stimulation, there was only a significant modulation of the power at the stimulation frequency, which was state-dependent (eyes-closed state: MI = 0.043 ± 0.015; *p* = 0.005; eyes-open state: MI = -0.014 ± 0.013; *p* = 0.282, difference *p* = 0.005).

Together, the data from the three participants demonstrate modulation of endogenous cortical oscillations by 10 Hz stimulation during and after stimulation, depending on behavioral state. In particular, the effects on endogenous oscillation that outlasted stimulation followed the predictions of the network model as well as the summation model. In agreement with entrainment of cortical oscillations by periodic brain stimulation, we found a shift toward the stimulation frequency for P001 in the task-engaged state. However, we also found enhancement of power at the endogenous frequency, which is hard to reconcile with entrainment (for example, in P001 after stimulation and in P005 during and after stimulation).

The effect of stimulation on endogenous oscillations, during stimulation in particular, observed in participants P001 and P005 supports the observations from the static summation model. In the eyes-closed state, during which alpha oscillations are strongest, stimulation produced little change, while in the eyes -open state, stimulation enhanced oscillation power in the endogenous frequency. In contrast, in the task-engaged state, stimulation enhanced power in the stimulation frequency, suggesting stimulation produced entrainment (or 1:1 frequency locking). The data from P008 does not fully agree with the summation model, as enhancement was observed in the stimulation frequency in both the eyes-open and eyes-closed states; the stimulation frequency being close to the first harmonic of the endogenous frequency is a potential confounding variable that could explain this difference.

The predictions from the network model are confirmed by the results from participants P001 and P005. However, in contrast to the model that predicts enhancement in power at stimulation as well as endogenous frequencies in the eyes-open state, the data from participant P005 showed no change in power at stimulation frequency. There are multiple possible reasons for this discrepancy between the experimental data and model. For example, the specific locations of stimulation electrodes in the experiment were more widespread and not just limited to the recording site, as they were in our model by definition. Indeed, we found that enhancement at 10 Hz was present, in average, across the participants, but that it was only observed local to the stimulation electrodes (S5 Fig) and reflected in the statistics of modulation indices as well (Fig 4B).

## Discussion

We elucidated the effects of 10 Hz stimulation on cortical network dynamics to advance the mechanistic understanding of interaction between cortical oscillations and periodic brain stimulation. We leveraged the unique access to human cortex in patients with medication-refractory epilepsy to provide experimental support. We attribute the effects that outlast stimulation to cortico-cortical network synchronization and the state-dependent effect to the thalamo-cortical network. Previous simulations of large-scale cortical networks with physiological propagation delays have demonstrated outlasting effects resulting from inherent multistable behavior mediated by the propagation delays in the long-range connections [8,20]. Similar cortico-cortical interactions may play a further role in the dynamics reported here. The choice of components in our model was motivated by biological plausibility. The thalamo-cortical network is the generator of alpha oscillations, and the cortico-cortical network involvement is motivated by the abundance of recurrent connections in the cortex. Although our model is a simplistic approximation of these circuits, the behavior of the model successfully reflected the dynamics observed in ECoG data. Moreover, the dynamics of the recurrent loop determines the frequency of oscillation exhibited by the network. Hence, this model can be applicable to oscillations other than alpha oscillations and networks other than the thalamo-cortical network, provided there exists a loop that mediates recurrent excitation. It is important to note that, although our model supports the experimental observations based only on network interactions, other studies have suggested the possibility that synaptic plasticity also plays a role [21,28]. It is possible that the stimulation duration was not long enough to recruit plasticity in our study. Typically, stimulation is applied in the order of minutes in studies in which outlasting effects are observed in stark contrast to the short 5 s segments used here. Thus, the time scale of observation is likely shorter than the time scale in which plasticity-induced changes could be observed. This restricts the comparison with models and experiments that study plasticity in the context of transcranial current stimulation.

In contrast to previous experimental studies, we recorded from the cortical surface while directly stimulating the cortical tissue. This approach helped us avoid issues of spatial filtering and artifact contamination observed in noninvasive stimulation and recording approaches. In agreement with previously postulated mechanisms [23,27], we found differential modulation of cortical oscillations by 10 Hz stimulation as a function of the endogenous state of the stimulated networks. In the eyes-closed state, stimulation had little effect on the oscillation dynamics. In the eyes-open state, the stimulation altered power at the endogenous frequency. In contrast, in the task-engaged state, the reduced endogenous peak permitted a response matched to the stimulation frequency. Our data demonstrate that cortical oscillations can indeed be enhanced by rhythmic stimulation and that even short periods of stimulation can have effects that, at least briefly, outlast stimulation.

Our results contribute to an ongoing debate about whether network entrainment or plasticity mediates the outlasting effects of periodic brain stimulation. The former hinges on the idea that periodic stimulation causes a realignment of phase of the oscillators resulting in an increase in oscillation strength at the stimulation frequency [35]. Our observation that the oscillation power was enhanced at the stimulation frequency and not at the endogenous frequency during the task-engaged state demonstrates the presence of entrainment. The entrainment hypothesis has been explored in studies that employed tACS stimulation and EEG [9,27,36], and results with varying degrees of support for this hypothesis have been obtained. In contrast, recent studies failed to find evidence of entrainment as a mechanism for oscillation power increases [28,29]. The studies employed short duration stimulation (comparable to the duration used in our study) and found that there was no evidence for entrainment, despite the observed outlasting effects. Hence, the studies argue plasticity to be the underlying mechanism. In our study, we found signs of entrainment (during the task-engaged state) as well as signs of enhancement of the endogenous oscillation (eyes-open condition). Therefore, the debate on the presence of entrainment could potentially be resolved by recasting it in the framework of state-dependent effects that we have demonstrated here. In fact, state-dependent effects have also been observed in tACS studies employing stimulation at frequency bands other than alpha band [37]. Our summation model proposes that the strength of the stimulation relative to the strength of the oscillation is an important contributing factor for the state-dependent effect. An alternate explanation for this effect is that weak network oscillations are generally more malleable to weak stimulation than strong network oscillations due to a ceiling effect. Specifically, strong oscillations have been suggested to correspond to a scenario in which all endogenous oscillators are already synchronized and, accordingly, no further entrainment by stimulation would be possible [27].

Our findings demonstrate that stimulation in the alpha frequency band may differ in its effect as a function of the endogenous state during stimulation. Of note, the functional implications of such differential modulation at varying frequencies within the alpha frequency band remain unclear. Furthermore, stimulation at matched frequencies, as used in individualized alpha frequency stimulation (IAF) for tACS, may reveal different response dynamics. Data from P008 supports this hypothesis, as an endogenous oscillation was observed at a frequency that was a subharmonic of stimulation frequency. Enhancement was observed at stimulation frequency as well as at the endogenous frequency irrespective of whether the participant’s eyes were closed or open.

The fact that power at 10 Hz increased locally (<20 mm; S5 Fig) suggests that it is indeed possible to enhance oscillation power at the stimulation frequency. It is likely that the strength of stimulation relative to endogenous oscillation was higher locally, and that this resulted in a scenario similar to task-engaged state predicted by the simple summation model. The strength of stimulus relative to ongoing oscillation is an important parameter in designing studies and is seldom studied in humans due to various limitations. Our dataset is limited to a single stimulation strength, which makes such analysis difficult, and further studies incorporating multiple stimulation strengths are required. Additionally, the spatial effect of stimulation is important for studies that attempt to address functions that are localized anatomically. Many tACS studies utilize large electrodes to produce an effect over a wide region. When the function under investigation is localized to anatomically specific regions, it might be advisable to utilize electrode configuration that produces a localized electric field as close as possible to the anatomical region.

Direct cortical stimulation is widely used in clinical settings, albeit in a different context. Developed by Wilder Penfield in the 1950s, it is generally used for localizing language areas and sensorimotor cortex (eloquent cortex) for surgical resection planning in patients suffering from seizures [30,31]. The approach involves using biphasic pulses at frequencies higher than were used in this study (50 Hz versus 10 Hz) to produce reversible, temporary microlesions in the stimulated site. These microlesions help to identify the functions associated with the areas [38–43]. However, the electrographic data obtained during stimulation are seldom analyzed, due to severe stimulation artifacts. Alternately, using low frequency stimulation allows for analysis of stimulation effects. Utilizing this strategy, cortico-cortical evoked potentials (CCEPs) induced by low frequency stimulation (1 Hz) have been used to map functionally connected areas [44–49]. Apart from these, chronically implanted devices that use electrical stimulation for termination of seizures have also been developed [50]. In the future, this approach can be extended to study the neural and behavioral responses to different frequencies. An added advantage of direct cortical stimulation (at low amplitudes) is that there are no perceptual effects that may confound the effect of stimulation in studies of noninvasive brain stimulation.

Despite the clear advantages of our approach based on ECoG recordings and stimulation, there are also important technical limitations. First, the actual waveforms of rTMS and tACS, even though both have a 10 Hz structure, differ from the pulsed waveform used in our study. Consequently, the effects of stimulation on neuronal tissue are different. tACS is believed to produce sub-threshold modulation with stimulation current flowing perpendicular to the cortical surface. In contrast, rTMS produces supra-threshold modulation with current flowing tangential to the cortical surface [51]. Like rTMS, biphasic stimulation produces a local supra-threshold modulation. Our choice of biphasic pulses was motivated by the stimulator hardware and stimulation waveforms approved for direct cortical stimulation in human participants. As a benefit of the waveform used in our study, artifact removal was simplified. Only a brief period of the raw signal during and immediately after the pulse was contaminated with a stimulation artifact. In spite of the above differences, we argue that the key shared feature between all stimulation waveforms is the periodic, 10 Hz structure. Second, as for any ECoG study, our results were obtained from patients with severe epilepsy a few days after head surgery for electrode implantation. Although we excluded electrodes that were in proximity to the clinically determined epileptic focus, the responses obtained may differ from the healthy intact brain, as patients with focal seizures often exhibit abnormal global oscillation patterns [52]. Finally, given the small sample sizes and variability in electrode locations and stimulation locations in these patients, ECoG data tends to be more heterogeneous than data from other electrophysiology techniques. This was also the case here. Nevertheless, as argued before, the opportunity to measure directly from cortical surface help us to elucidate interaction between stimulation and endogenous dynamics at a finer spatial and temporal scale.

In conclusion, we provide new mechanistic models supported by invasive human electrophysiological recordings for the modulation of endogenous oscillations by periodic brain stimulation. Despite the limitations discussed above, our findings carry important implications for the design and interpretation of brain stimulation studies. This is of heightened importance, given the emergence of novel therapeutic brain stimulation paradigms that target alpha oscillations [17,18]. First, it is highly recommended that the state of the participant is uniform within a study and that there is independent verification of whether the according instructions to the participant were followed. Second, if an enhancement of endogenous alpha oscillations is (clinically) desired, stimulation during the eyes-open state may be the most effective approach. Third, if an increase (or likely change in general) of the alpha frequency is desired, stimulating in a state of suppressed alpha oscillations (e.g., state of heightened behavioral arousal or attention) likely provides the best state for stimulation. Fourth, the response to stimulation may vary from participant to participant and (ideally simultaneous) electrophysiological monitoring is essential. We argue that the study of these principles, together with the further development of computational and mathematical models, will advance brain stimulation toward becoming a clinically effective modality for the restoration of functional alpha oscillations, perhaps the most fundamental electric activity pattern generated by the brain [2].

## Materials and Methods

### ECoG Data Collection and Direct Cortical Stimulation

All procedures in this study were approved by the Institutional Review Board of the University of North Carolina at Chapel Hill (IRB Number 13–2710), and written informed consent was obtained from all the participants. The participants underwent temporary implantation of subdural electrodes for presurgical localization of seizure focus in the Epilepsy Monitoring Unit at the UNC Neurosciences Hospital. Electrode grids were implanted over the different cortical regions as described in Table 1. S2 Fig denotes the location of electrodes for the three patients.

**Table 1:** Clinical Information of Participants.

Participant	Age	Sex	Handedness	Clinical Seizure Focus	Grid Locations (Number of Electrodes)	Location of Stimulation Electrodes (Number of Electrode Pairs Stimulated)	Total Number of Stimulations (Stimulation per Electrode Pair per Condition)
P001	21	F	R	Right Frontal Lobe	Right Frontal Lobe (29), Right Parietal Lobe (24), Right Limbic Lobe (3)	Right Anterior Frontal Lobe (8), Right Parietal Lobe (6)	28 (1)
P005	30	F	R	Unknown Seizure Focus	Left Frontal Lobe (40), Left Temporal Lobe (38), Left Parietal Lobe (12), Left Limbic Lobe (3)	Left Frontal Lobe (2), Left Parietal Lobe (1), Left Temporal Lobe (1)	18 (2)
P008	23	F	R	Bitemporal Lobe	Bilateral Parahippocampal Gyrus (16), Left Frontal Lobe (12), Right Frontal Lobe (12), Left Parietal Lobe (6), Right Parietal Lobe (4), Left Temporal Lobe (14), Right Temporal Lobe (4)	Left Parietal Lobe (3), Right Parietal Lobe (2)	20 (2)

The electrodes were made of platinum-iridium alloy, were 4 mm in diameter (2.5 mm exposed), and were embedded in silicone (Ad-Tech Medical, Racine, Wisconsin, United States). The inter-electrode distance in each grid was 10 mm. Four electrodes in a separate set placed far from the recording grids were chosen to be reference electrodes. ECoG signals were recorded using a 128-channel Aura LTM 64 acquisition system and the corresponding TWin software (Grass Technologies, Warwick, Rhode Island, United States) at 800 Hz sampling rate.

Electrical stimulation consisted of a train of biphasic pulses 2 mA in amplitude and 200 μs in duration, with a pulse every 100 ms (10 Hz) generated by a S12x cortical stimulator (Grass Technologies, Warwick, Rhode Island, United States) and applied between a pair of adjacent electrodes for 5 s (Electrodes marked Blue in S2 Fig). The stimulations were spaced at about 15 s between trials to enable safety monitoring for the occurrence of after-discharges.

### Behavioral Tasks

The experimental paradigm consisted of three conditions. In the first condition, the participants were asked to close their eyes and relax. This was called the eyes-closed state. In the second condition, the participants were asked to open their eyes and relax without focusing on anything. This was named the eyes-open state. In the third state, the task-engaged state, the participants performed a visual working memory task based on the task developed by Luck and Vogel [53]. Data was acquired from participants P005 and P008 in both the eyes-closed and eyes-open conditions but not during the working memory task. Participant P001 completed the eyes-closed task and the working memory task. The task was programmed in Matlab using Psychophysics Toolbox. The task required the participant to look at a set of colored dots for 1.5 s and indicate with a key press whether or not there was any change in color in the set of dots presented in a second test set 0.9 s later (memory period with no stimuli on the screen). This task required considerable cognitive effort from the participant. The participant was able to detect the change accurately in 71% of trials with a reaction time of 909.80 ± 61.52 ms.

### Data Analysis

The collected ECoG data was transferred from the clinical acquisition system to a research workstation in EDF format [54]. Switching circuits designed to protect the amplifier during stimulation prevented recording of data from stimulating electrodes, and, hence, data from stimulating electrodes were not included in the analysis. Electrodes over epileptogenic tissue were identified from clinical seizure traces and then excluded from further analysis. Additionally, electrodes that showed significant noise were also excluded (four electrodes in P001). Each stimulation trial was identified (manually by visual inspection for stimulation artifacts) and marked in the open source software EDFBrowser. Later, the data were processed to remove stimulation artifacts and line noise using custom scripts written in Matlab (Mathworks Inc.). A detailed description of the stimulation artifact removal algorithm is provided below. Following artifact removal, line noise was removed using a second order IIR notch filter. Then, the signals were re-referenced to a common average reference. Signal power spectra were calculated at 0.1 Hz resolution between 0.5 Hz and 20 Hz and at 1 Hz resolution between 21 Hz and 80 Hz by convolving with Morlet wavelets of corresponding frequencies. Modulation indices were computed to quantify changes in power at specific frequency bands in different epochs. Mathematically,

$$Modulation\;Index\;=\frac{\left(S_{b}^{2}-\;S_{b}^{1}\right)}{\left(S_{b}^{2}+S_{b}^{1}\right)}$$

where $S_{b}^{1}$ and $S_{b}^{2}$ are average power in frequency band *b* in epochs 1 and 2. In the analysis, the 5 s window before stimulation was chosen as epoch 1, and the 5 s windows during stimulation and after stimulation were chosen as epoch 2. A symmetric 3 Hz band was chosen around the peak of the spectra for the endogenous frequency, and a symmetric 2 Hz band was chosen around 10 Hz for the stimulation frequency. The resulting measure was bounded between -1 and 1 and amenable to comparison.

### Statistics

Statistics included one sample *t* tests to determine the significance of modulation indices and two sample *t* tests for determining significance of differences in modulation indices between the two states under investigation. All statistical analysis was performed using Matlab Statistics Toolbox (Mathworks Inc.).

### Stimulation Artifact Removal

Stimulation artifacts appeared as transient deflections (approximately 10 ms in duration) in traces recorded from electrodes near the stimulating electrodes. A template matching algorithm was used to remove these artifacts. First, the waveforms were upsampled to 3,200 samples/second (anti-alias filtering followed by linear interpolation), as this allowed more robust estimation of these deflections’ peaks at later steps. The next step involved determination of the artifacts’ temporal location. By comparing the power around 100 Hz (corresponding to the artifact waveform), the electrode with the highest stimulation artifact amplitude was determined. Then, the trace recorded from the chosen electrode was high pass filtered to remove low frequency biological signals using a cubic polynomial fitting algorithm [55]. Artifacts were detected using a threshold crossing approach, and corresponding times were collected. The artifacts corresponding to a stimulation trial occurred at all electrodes at the same time point. Hence, the times collected from the single channel provided the temporal location of artifacts in all electrodes. The next step of the algorithm was to remove the detected artifacts. Waveforms of artifacts were extracted in a temporal segment around the detected location and aligned to their peaks. For each artifact, a template was constructed by averaging the waveforms of five artifacts (the current waveform, two preceding waveforms, and two following waveforms). Next, the template was scaled to the amplitude of the artifact and subtracted from the trace. This resulted in offsets at the edges of the temporal segments. These abrupt discontinuities were removed by subtracting the linear interpolation between the offsets from the segment, which resulted in a continuous waveform. Finally, the signals were downsampled back to 800 Hz after anti-alias filtering.

### Extraction of Electrode Location from Neuroimaging Data

3D Slicer [56] was used to analyze and extract electrode locations from CT images obtained after implantation of subdural electrodes. A multistep procedure was followed to generate images showing activation of cortical surface. The postoperative MRI was coregistered to postoperative CT in Slicer. Skull stripping was performed using ROBEX [57], and the gray matter and white matter were then segmented using ITK-Snap [58]. The surface model was generated using Slicer, and the model was imported into Matlab. The anatomical locations of the electrodes were determined by coregistering the MRI Image to the MNI Atlas [59], recomputing electrode locations in the MNI space, transforming these locations to Talairach space, and using the Talairach Client [60] to obtain the label of the gray matter nearest to the coordinate representing electrode location. The shape and anatomical landmarks of participant P005’s brain deviated significantly from the atlas MRI. Hence, we estimated the anatomical locations based on patient MRI directly instead of patient MRI coregistered to atlas MRI.

### Computational Model

To generate mechanistic hypotheses that can explain our experimental findings, we used a population-scale neural oscillator network model, which combines essential components of cortical anatomy as well as interactions between cortical and subcortical areas. To propose a functional mechanism underlying the effect after termination of stimulation, we deliberately chose a simplified network model inspired and adapted from existing population-scale cortical network models [61,62], in which we concentrated our attention on the role of feedback connections as opposed to local recurrent dynamics. It was designed primarily to investigate the influence of recurrence and feedback on both endogenous and stimulation-induced oscillations. The model consisted of N recurrently connected neural oscillators composed of excitatory (*e*) and inhibitory (*i*) units that spontaneously expressed oscillatory alpha activity due to delayed thalamo-cortical feedback. The membrane potential proxy vectors ***V***_***e***_(*t*) and ***V***_***i***_(*t*) obeyed the dimensionless equations

$$\begin{array}{l} \tau_{e}{\dot{V}}_{e}(t)=a\;V_{e}(t)+G_{e\to e}+G_{i\to e}+F_{1}+F_{2}+I_{bias}+I_{task}+\sqrt{2D}\xi_{e}(t)+S(t)\\ \tau_{i}{\dot{V}}_{\iota }(t)=a\;V_{i}(t)+G_{e\to i}+G_{i\to i}+\sqrt{2D}\xi_{i}(t)+S(t) \end{array}$$

The terms ***ξ***_***e***,***i***_ referred to independent, zero mean Gaussian white noise processes. The synaptic time constants of excitatory and inhibitory populations were given by *τ*_*e*_ and *τ*_*i*_, respectively, and the membrane leakage coefficient was *a*. Recurrent inputs from population *n* = *e*, *i* to population *m* = *e*, *i* were defined by the following:

$$G_{n\to m}=g_{nm}\;W_{nm}\cdot f\left[V_{n}\left(t\right)\right],$$

where

$$f\left[V\right]={\left(1+\text{exp}\left[-35\left(V-h\right)\right]\right)}^{-1}$$

was a nonlinear saturating response function with threshold *h*.

Thalamo-cortical feedback, through which endogenous oscillatory activity emerged in the eyes-closed and eyes-open conditions, was defined as

$$F_{1}=g_{1}W_{1}\cdot f\left[V_{e}\left(t-D_{1}\right)\right].$$

Cortico-cortical feedback mediated by more distant cortical nets was defined as

$$F_{2}=g_{2}W_{2}\cdot f\left[V_{e}\left(t-D_{2}\right)\right].$$

For each of those components, ***W***_*nm*_ = (*ρN*)^−1^⋅**1**, ***W***_1_ = (*ρN*)^−1^⋅**1,** and ***W***_2_ = (*ρN*)^−1^⋅**1** represented sparse synaptic connectivity matrices with connection probability *ρ*. Feedback delays, representing conduction latencies to and from sub-cortical and other cortical areas, were given by *D*_1_ and *D*_2_, respectively. These were adjusted to fit the spectral features expressed by the experimental data. Task-dependent input to the excitatory units was scaled as a function of the condition: it was minimal in the eyes-closed condition, intermediate for the eyes-open state, and maximal in the task-engaged state. The stimulation ***S*** is applied to all neurons, defined as a biphasic pulse train with frequency of 10 Hz, intensity S for 200 *μs* and -S for another 200 *μs*, and then set to zero until the next cycle. Numerical integration was performed using a Euler-Maruyama scheme with time step of dt = 1 ms. Spectral analysis was performed over a synthetic ECoG signal defined by the network-wide potential average, i.e., *ECoG* = *N* ^−1^ ∑_*N*_[*V*_*e*_ + *V*_*i*_]. The parameter values used in the model are listed in Table 2.

**Table 2:** Model parameters.

Symbol	Value	Definition
*ρ*	0.8	Connection probability
*τ*_*e*_	10.0 ms	Excitatory synaptic time constant
*τ*_*i*_	6.6 ms	Inhibitory synaptic time constant
*a*	-1.5	Membrane leak constant
*g*_*ee*_	1.0	Synaptic gain *e* → *e*
*g*_*ei*_	1.0	Synaptic gain *e* → *i*
*g*_*ie*_	-1.0	Synaptic gain *i* → *e*
*g*_*ii*_	-1.0	Synaptic gain *i* → *i*
*g*_1_	-0.5	Thalamo-cortical feedback gain
*g*_2_	0.85	Large-scale cortico-cortical feedback gain
*h*_*o*_	-0.50	Recurrent interactions threshold
*h*_1_	-0.30	Thalamo-cortical feedback threshold
*h*_2_	0.15	Cortico-cortical feedback threshold
*D*_1_	65 ms	Thalamo-cortical delay
*D*_2_	290 ms	Cortico-cortical delay
*I*_*Bias*_	-0.23	Baseline input
*I*_*Resting*_	0.00	Resting state input
*I*_*Eyes*−*open*_	0.27	Input in the eyes-open condition
*I*_*task*_	0.50	Task-related input
*S*	0.20	Stimulation amplitude
*D*	0.01	Noise intensity

## Supporting Information

S1 Fig Differential roles of thalamo-cortical and cortico-cortical loops. (A) With thalamic interactions absent, no endogenous alpha oscillation was generated, and stimulation produced an increase in power at stimulation frequency in the eyes-open and task-engaged states without any outlasting effect. (B) Spectral dynamics of the model with cortico-cortical interactions absent revealed a lack of outlasting stimulation effects. In the simulated eyes-open state, the power at the endogenous frequency increased during stimulation but returned to prestimulation levels in the epoch immediately after stimulation. In the task-engaged state, stimulation caused an increase in power at the stimulation frequency only during stimulation.
(TIF)

S2 Fig Electrode locations. The figure shows electrode coverage over different regions for each of the three participants included in this study. Electrodes marked in blue denote electrodes that were both stimulated and recorded, while electrodes marked in red were the electrodes that were used only for recording. Participant P008 had 16 depth electrodes sampling parahippocampal gyri, which are not shown in this figure. These electrodes were not included in the analysis.
(TIF)

S3 Fig Stimulation induced 10 Hz oscillation. (A) Time-domain signal of a single trial from participant P001 showing stimulation artifacts in the raw data (top) and the significant reduction in artifact amplitude after artifact suppression (middle) as well as after re-referencing. The traces enclosed in black boxes are displayed in detail on the right, showing the 10 Hz oscillatory structure. (B) Spectrogram of a single trial showing temporal evolution of 10 Hz oscillation at stimulation onset and the corresponding time domain signal. It can also be seen that after stimulation offset, strong oscillation persists, albeit at a slightly lower frequency.
(TIF)

S4 Fig Stimulation induced enhancement at endogenous frequency. Temporal evolution of power spectra observed in an example electrode reveals enhancement at endogenous frequency during stimulation (orange arrow) as well as after stimulation (blue arrow) in eyes-open condition.
(TIF)

S5 Fig Effect of distance on modulation index. During stimulation, the power at stimulation frequency was enhanced very close to stimulation electrodes (<20 mm) while there was no change at longer distances (>20 mm).
(TIF)

## Abbreviations

CCEP: cortico-cortical evoked potential
ECoG: electrocorticography
EEG: electroencephalogram
FDA: Food and Drug Administration
IAF: individualized alpha frequency
MI: modulation index
rTMS: repetitive transcranial magnetic stimulation
tACS: transcranial alternating current stimulation
