# Supplementary figures and images for: Modulation of Cortical Oscillations by Low-Frequency Direct Cortical Stimulation Is State-Dependent
Source: PLoS Biol. 2016 Mar 29;14(3):e1002424. doi: 10.1371/journal.pbio.1002424 (PMC4811434; doi:10.1371/journal.pbio.1002424)

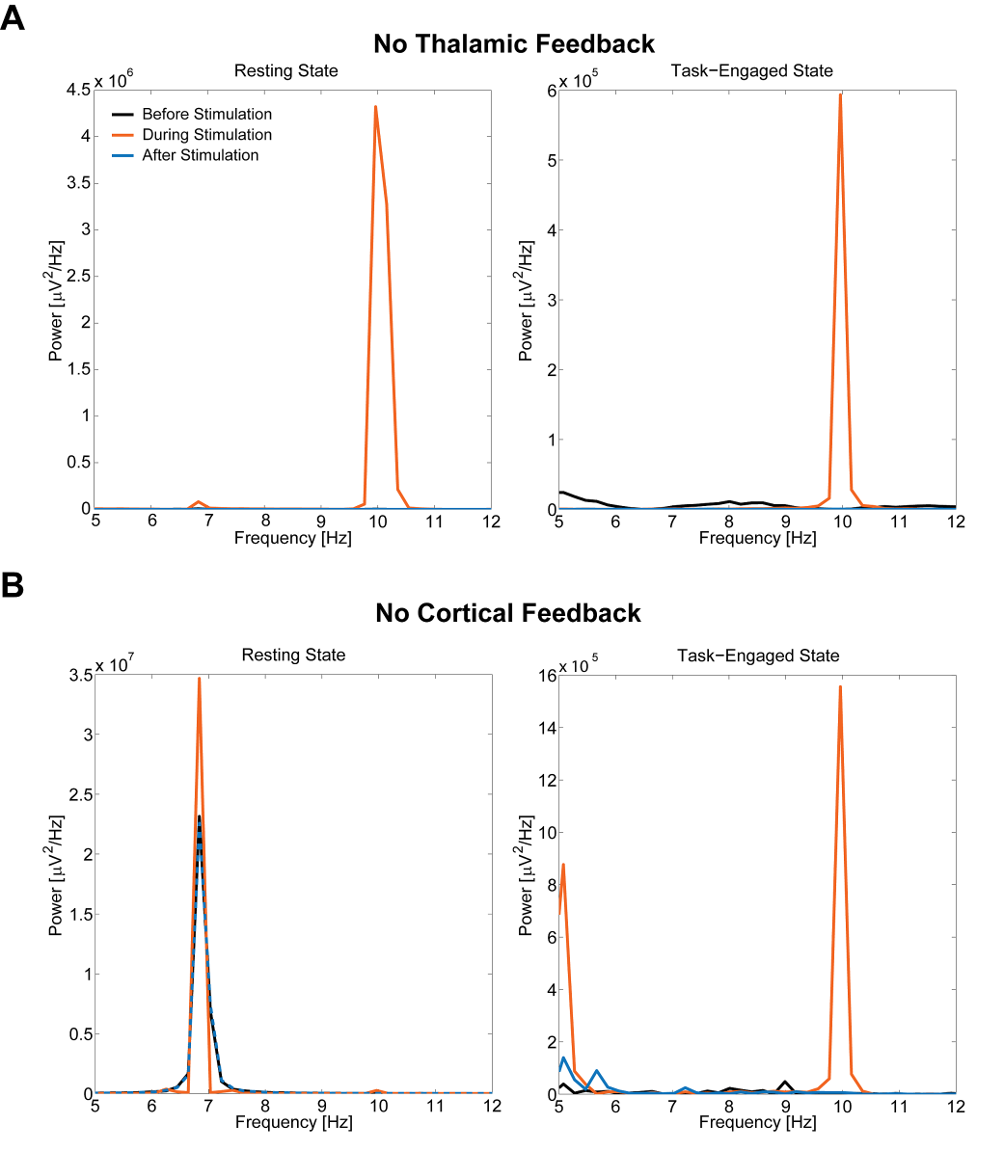

Supplement: S1 Fig — (A) With thalamic interactions absent, no endogenous alpha oscillation was generated, and stimulation produced an increase in power at stimulation frequency in the eyes-open and task-engaged states without any outlasting effect. (B) Spectral dynamics of the model with cortico-cortical interactions absent revealed a lack of outlasting stimulation effects. In the simulated eyes-open state, the power at the endogenous frequency increased during stimulation but returned to prestimulation levels in the epoch immediately after stimulation. In the task-engaged state, stimulation caused an increase in power at the stimulation frequency only during stimulation. (TIF) [file pbio.1002424.s001.tif]

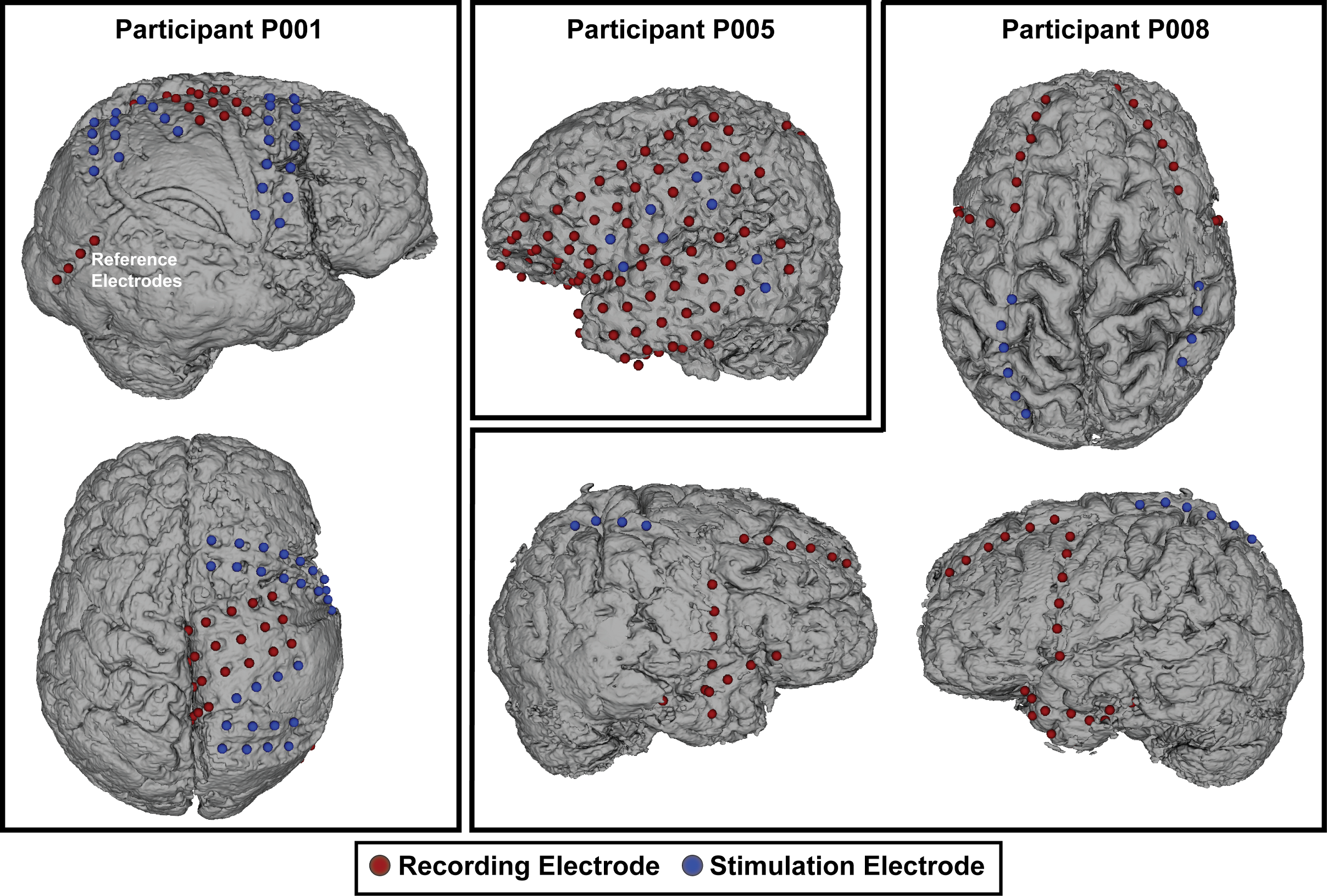

Supplement: S2 Fig — The figure shows electrode coverage over different regions for each of the three participants included in this study. Electrodes marked in blue denote electrodes that were both stimulated and recorded, while electrodes marked in red were the electrodes that were used only for recording. Participant P008 had 16 depth electrodes sampling parahippocampal gyri, which are not shown in this figure. These electrodes were not included in the analysis. (TIF) [file pbio.1002424.s002.tif]

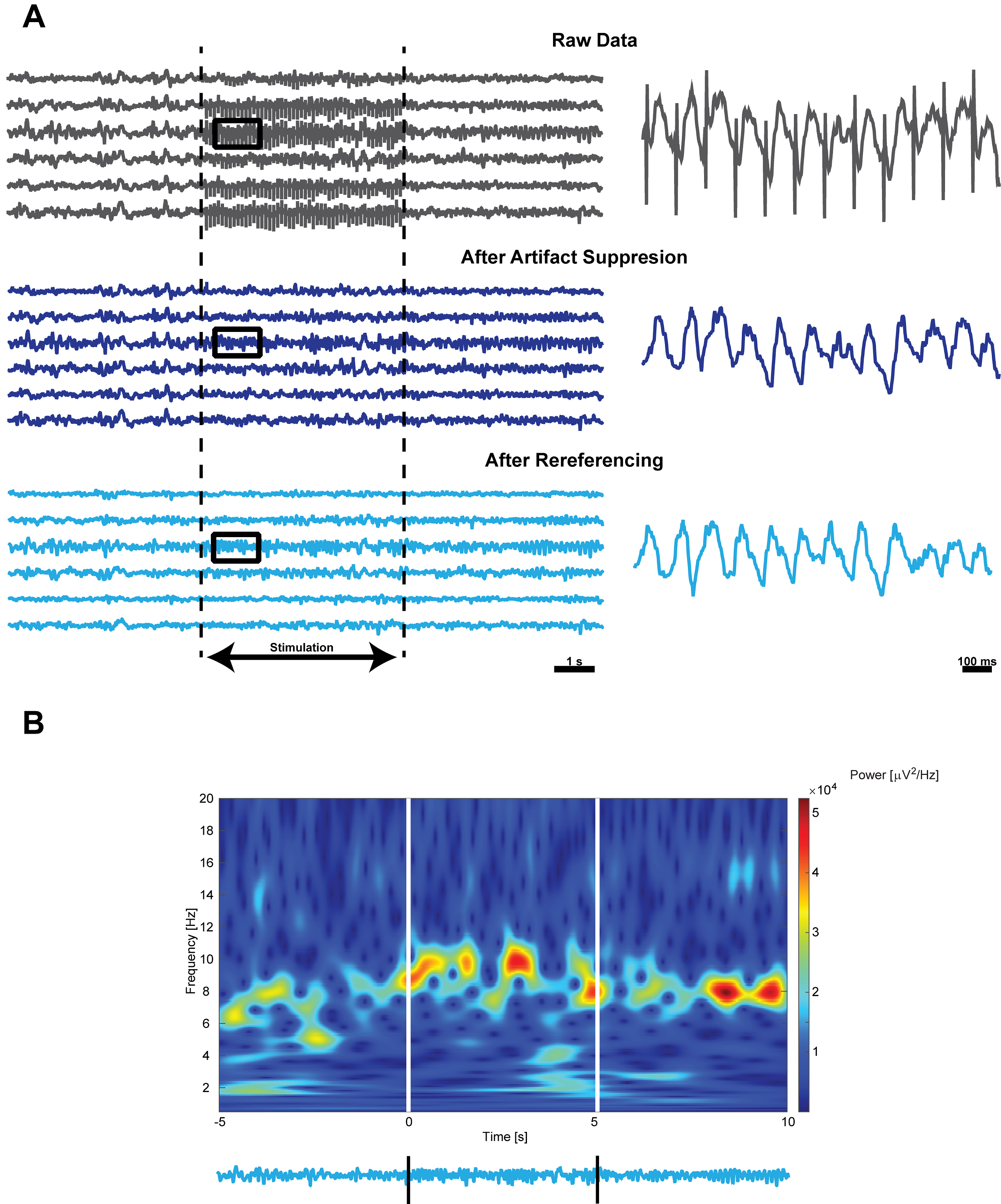

Supplement: S3 Fig — (A) Time-domain signal of a single trial from participant P001 showing stimulation artifacts in the raw data (top) and the significant reduction in artifact amplitude after artifact suppression (middle) as well as after re-referencing. The traces enclosed in black boxes are displayed in detail on the right, showing the 10 Hz oscillatory structure. (B) Spectrogram of a single trial showing temporal evolution of 10 Hz oscillation at stimulation onset and the corresponding time domain signal. It can also be seen that after stimulation offset, strong oscillation persists, albeit at a slightly lower frequency. (TIF) [file pbio.1002424.s003.tif]

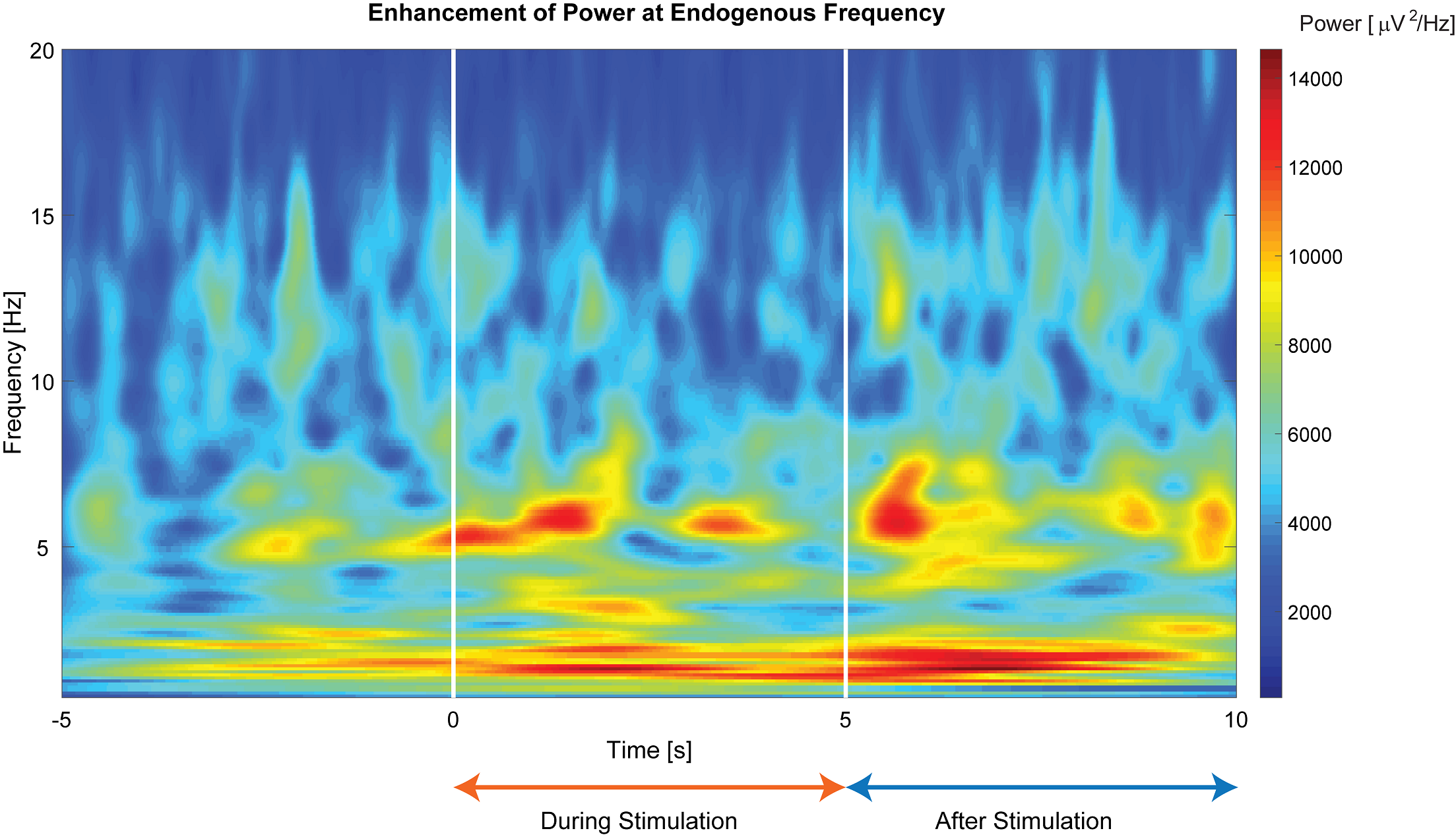

Supplement: S4 Fig — Temporal evolution of power spectra observed in an example electrode reveals enhancement at endogenous frequency during stimulation (orange arrow) as well as after stimulation (blue arrow) in eyes-open condition. (TIF) [file pbio.1002424.s004.tif]

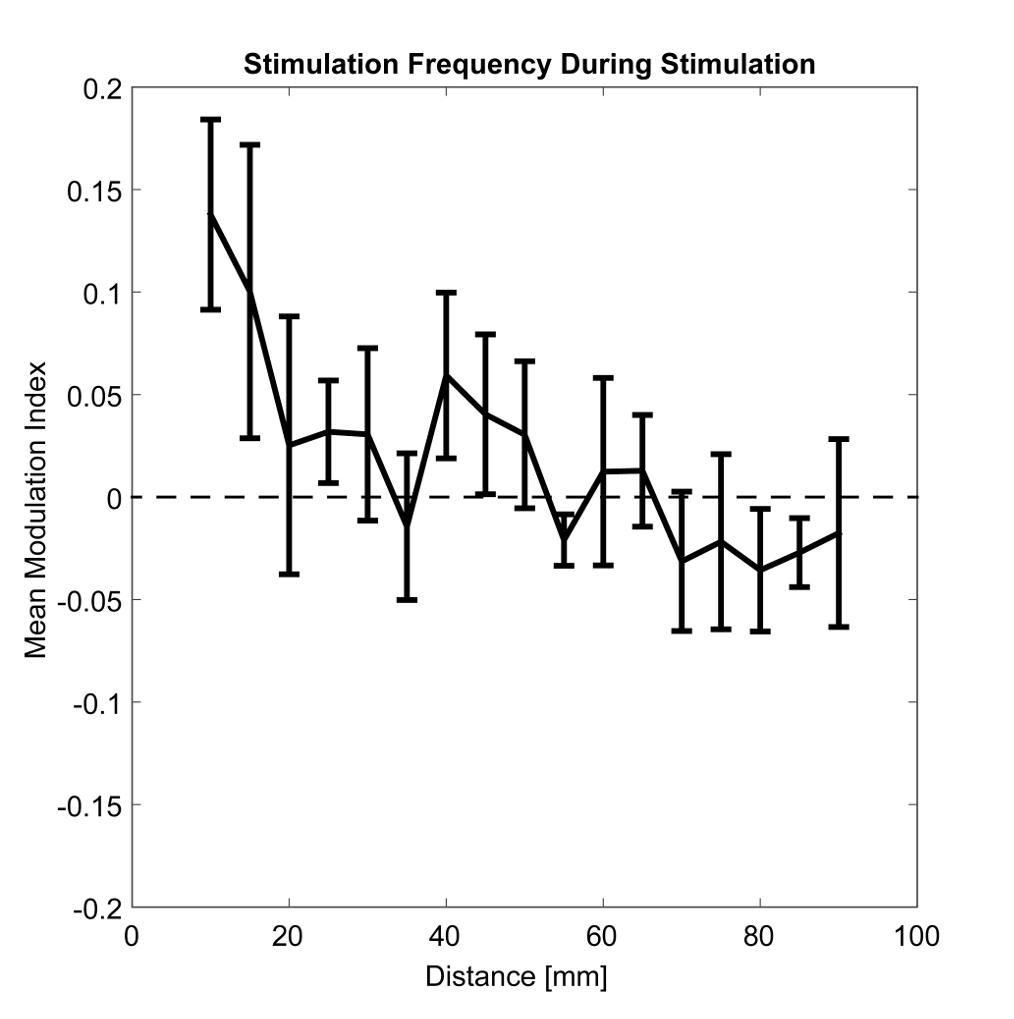

Supplement: S5 Fig — During stimulation, the power at stimulation frequency was enhanced very close to stimulation electrodes (<20 mm) while there was no change at longer distances (>20 mm). (TIF) [file pbio.1002424.s005.tif]
